# The Impact of Maternal Education on Neonatal Outcomes in Preeclamptic Pregnancies from a Low-Resource Settings

**DOI:** 10.3390/jcm14113937

**Published:** 2025-06-03

**Authors:** Victor Bogdan Buciu, Denis Mihai Șerban, Sebastian Olariu, Dorin Novacescu, Cosmin Cîtu, Sebastian Ciurescu, Larisa Tomescu, Adrian Claudiu Rațiu, Ioan Sas, Mihai Ionac, Veronica-Daniela Chiriac

**Affiliations:** 1 Doctoral School, “Victor Babes” University of Medicine and Pharmacy Timisoara, E. Murgu Square, No. 2, 300041 Timisoara, Romania; victor.buciu@umft.ro (V.B.B.); raul.olariu@umft.ro (S.O.); sebastian.ciurescu@umft.ro (S.C.); 2 Department of Obstetrics-Gynaecology, Discipline of Obstetrics-Gynecology, “Victor Babes” University of Medicine and Pharmacy Timisoara, E. Murgu Square, No. 2, 300041 Timisoara, Romania; citu.ioan@umft.ro (C.C.); tomescu.larisa@umft.ro (L.T.); ratiu.adrian@umft.ro (A.C.R.); sas.ioan@umft.ro (I.S.); chiriac.veronica@umft.ro (V.-D.C.); 3 Department of Microscopic Morphology, “Victor Babes”, Discipline of Histology, Victor Babes” University of Medicine and Pharmacy Timisoara, E. Murgu Square, No. 2, 300041 Timisoara, Romania; novacescu.dorin@umft.ro; 4 Department of Microsurgery, Vascular Surgery and Scientific Research Methodology, “Victor Babes” University of Medicine and Pharmacy Timisoara, E. Murgu Square, No. 2, 300041 Timisoara, Romania; mihai.ionac@gmail.com

**Keywords:** preeclampsia, education, ISCED, neonatal outcomes, fetal growth restriction

## Abstract

**Background/Objective:** Preeclampsia is a hypertensive disorder associated with pregnancy that has a significant impact on maternal and neonatal health and has the potential to result in significant perinatal adverse outcomes. Maternal education has been proposed as a protective factor during pregnancy; however, its role in preeclamptic pregnancies remains unclear. This study aimed to explore the relationship between maternal education level, as defined by ISCED classification, and neonatal outcomes (birth weight, gestational age, and APGAR score) in pregnancies complicated by preeclampsia. **Methods:** A retrospective case-control analysis was conducted on 674 deliveries at a single tertiary center in Western Romania between January 2022 and August 2024. Neonatal outcomes, specifically birth weight, gestational age, and APGAR scores were studied and stratified into three ISCED-based maternal education subgroups. Statistical analyses, including ANOVA, chi-square tests, and logistic regression, were used to analyze the effect of maternal education, with confounders such as maternal age and chronic hypertension being controlled for. **Results:** Preeclampsia was associated with lower birth weight (*p* < 0.001), gestational age at birth (*p* < 0.001), and APGAR scores (*p* < 0.001) than the control group. Maternal level of education was associated with better neonatal outcomes in the preeclamptic group, with lower odds of fetal growth restriction (OR = 0.68, *p* = 0.03) and preterm birth; however, the effect was less pronounced in the control group. **Conclusions:** Maternal education partially mitigates the adverse effects of preeclampsia on neonatal well-being, birth weight, and gestational age at birth. These findings underscore the importance of incorporating maternal education into prenatal care programs to improve perinatal outcomes, with a special focus on high-risk pregnancies.

## 1. Introduction

Preeclampsia is a hypertension-induced disease that usually debuts after 20 weeks of gestation. It is characterized by elevated blood pressure, accompanied by proteinuria or another organ-specific sign of distress. Preeclampsia has a significant impact on both maternal and fetal health, with increased morbidity and mortality rates throughout the world [1]. Preeclampsia affects between 2% and 8% of all pregnancies and is a leading cause of maternal and perinatal complications [2]. Both preeclampsia and eclampsia result in more than 50,000 maternal deaths annually, with wide regional variations [3,4]. As recognized by the World Health Organization (WHO), hypertensive disorders during pregnancy are reported to account for nearly 10% of all maternal deaths worldwide [5].

In terms of public health, the WHO recognizes inequality in the delivery of prenatal care as a risk factor for preeclampsia, particularly in low- and middle-income countries, where both maternal and healthcare literacy are low [6]. The WHO guidelines emphasize the need for maternal education programs in prenatal care to improve outcomes and prevent the development of hypertension-related complications [5].

In Romania, the prevalence of preeclampsia is estimated to be between 4% and 7%, consistent with rates observed in other Eastern European countries [6]. However, significant regional disparities exist, particularly in rural and underserved populations, where access to specialized prenatal care and educational resources is limited. These disparities are especially relevant given the growing evidence that socioeconomic and educational factors influence the risk and management of hypertensive disorders during pregnancy.

Although the ISCED classification reflects formal educational attainment, it may also serve as a proxy for maternal health literacy, influencing women’s ability to recognize warning signs, adhere to medical advice, and seek timely prenatal care.

Previous studies have demonstrated that lower maternal education is associated with adverse birth outcomes, such as low birth weight and preterm birth [7,8]. However, it remains unclear whether education can mitigate these risks in the specific context of pregnancies complicated by preeclampsia. To date, no study has explored this association in Romania, a country with persistent regional disparities in education and access to prenatal care services.

Maternal education plays a key role in shaping pregnancy outcomes beyond the availability of prenatal care. Higher educational levels are related to higher health literacy, which can further lead to better food choices, adherence to prenatal indications, and improved decision-making regarding medical follow-up [7,8]. Understanding the impact of education of the mother on the well-being of the newborn in preeclamptic pregnancies can help develop prevention strategies, especially in populations where educational disparities exist.

Oxidative stress and inflammation are major causes of preeclampsia-related endothelial dysfunction [9]. In addition, anti-angiogenic factors, such as soluble fms-like tyrosine kinase-1 (sFlt-1), are involved in disease progression. These offer alternatives for biomarker-based diagnostic and treatment strategies [10]. Preeclampsia has also been linked to an increased risk of long-term postpartum cardiovascular complications. These include chronic hypertension, ischemic heart disease, and stroke in both mothers and children [11]. Neonatal respiratory adaptation is also affected, with newborns showing a higher incidence of respiratory distress syndrome and bronchopulmonary dysplasia [12]. While previous research suggests that higher education can enhance adherence to medical visits, encourage early prenatal show-up, and elevate belief in medical professionals [13], less is known about whether maternal education can reduce disease-related risks in preeclamptic pregnancies.

This study aims to assess whether maternal education level (ISCED), is proportionally associated with neonatal outcomes in pregnancies complicated by preeclampsia. Understanding this relationship may inform targeted educational and clinical interventions for at-risk populations, particularly in settings such as Romania, where educational disparities are pronounced.

## 2. Materials and Methods

### 2.1. Study Design

This retrospective case-control study was carried out in a single hospital in Western Romania. The dataset included 674 deliveries between January 2022 and August 2024, with 324 cases complicated by preeclampsia and 350 control cases without it. The primary objective of this study was to assess the impact of maternal education, stratified using the International Standard Classification of Education (ISCED) [14], on fetal birth weight, gestational age, and APGAR scores in pregnancies affected by preeclampsia. The above-mentioned ISCED were divided into three groups: low education (ISCED 1 and 2), medium education (ISCED 3 and 4), and high education (ISCED 5 and 6). We hypothesized that higher maternal education levels are linked to improved neonatal outcomes, even in the presence of the studied disease.

A sample size of at least 300 cases and 300 controls was estimated based on the prior incidence rates of preeclampsia and an expected moderate effect size (Cohen’s d = 0.4) on neonatal outcomes, with a statistical power of 0.8 and alpha = 0.05.

### 2.2. Ethical Considerations

The research protocol was reviewed and approved by the Ethics Committee of the University of Medicine and Pharmacy “Victor Babes” Timisoara, under approval number 78/02.10.2023. Informed consent was obtained from all participants in accordance with the principles of the Declaration of Helsinki. This study adhered to ethical guidelines, ensuring the confidentiality and voluntary participation of all subjects.

### 2.3. Inclusion and Exclusion Criteria

This study included singleton pregnancies with a gestational age of at least 24 weeks. Local guidelines dictate that pregnancies above 24 weeks of gestation should be considered viable. All patients were delivered at the study center between January 2022 and August 2024. To ensure a comprehensive analysis, only cases with available data on maternal education (ISCED) and neonatal outcomes (weight, gestational age, and APGAR scores) were included. The exclusion criteria included multiple pregnancies, congenital anomalies of the neonate or mother, missing or incomplete data for key variables, and severe maternal comorbidities, such as chronic renal disease or major cardiovascular conditions, which could incorrectly affect the study outcomes. Patients under 18 years of age were also excluded. To ensure comparability, the preeclampsia and control groups were matched using a nearest-neighbor strategy, making both groups homogeneous.

### 2.4. Data Collection

The data for this study were manually extracted by two medical doctors from the hospital’s perinatal registry using a standardized form in Microsoft Office Excel 2016. After data collection and review, 47 patients were excluded due to incomplete data. The key maternal variables included in the analysis were the presence or absence of preeclampsia, maternal age, and maternal weight. Neonatal characteristics, such as birth weight (g), gestational age (weeks of gestation), and APGAR scores, were also included. Low birth weight was defined as less than 2500 g, while fetal growth restriction (FGR) was classified as a birth weight below the 10th percentile. Hypertension was defined according to the American College of Obstetricians and Gynecologists (ACOG) criteria as a blood pressure reading of ≥140/90 mmHg or the use of antihypertensive medication [4]. Educational attainment was categorized using the ISCED [14] and divided into three subgroups according to the study design.

All data were anonymized and stored on encrypted institutional drives that were accessible only to the research team. Patient confidentiality was ensured throughout the study in compliance with the national data protection legislation and GDPR guidelines.

### 2.5. Statistical Analysis

Statistical analyses were performed using SPSS Version 26. Descriptive statistics were performed for maternal and neonatal characteristics, including means, ranges, and standard deviations for continuous variables. Proportions were determined for categorical variables like hypertension and education level (ISCED). For the primary analyses, independent *t*-tests were used to compare continuous variables between the preeclampsia and control groups, while chi-square tests were used for categorical variables. ANOVA was performed to assess differences in fetal outcomes across the ISCED subgroups. A *p*-value of less than 0.05 was considered statistically significant. To evaluate the effect of maternal education on neonatal outcomes, we used logistic regression, adjusting for potential confounders. Additionally, receiver operating characteristic (ROC) curves were used to assess the predictive value of ISCED level for birth weight and APGAR scores. The area under the curve (AUC) was calculated for each outcome to evaluate the discriminative power of maternal education.

## 3. Results

### 3.1. Characteristics of the Study Population

A total of 674 pregnancies were included in this study, of which 324 cases were complicated by preeclampsia and 350 control cases were without preeclampsia. A detailed descriptive analysis was performed to assess various factors, including maternal age, number of previous conceptions (gravidity), total number of viable fetuses carried (parity), fetal weight (g), and education level categorized by the ISCED. Gestational age at birth was recorded in completed weeks, as confirmed during postpartum evaluation by a specialist pediatrician. Additionally, birth weight (g), fetal length (cm), cranial circumference (cm), and abdominal circumference (cm) were measured at the same time. The Apgar score, a standardized assessment evaluating appearance, pulse, grimace, activity, and respiration, was recorded one minute after birth to assess the newborn’s immediate health status. All data assessed is presented in Table 1.

Gestational age at birth was significantly lower in the preeclampsia group, with a mean of 34 weeks compared to 38 weeks in the control group (*p* < 0.001), reinforcing its strong association with preterm birth. Maternal age showed no significant difference, with mean values of 29.26 and 29.05 years, respectively (*p* = 0.640). Similarly, the number of conceptions did not differ significantly (2.45 vs. 2.61, *p* = 0.170), but the number of viable fetuses carried was lower in the preeclampsia group (1.72 vs. 2.04, *p* = 0.002), suggesting a link to a reduced ability to carry viability or to a viable birth. Fetal growth was notably impacted, with preeclampsia-exposed neonates showing significantly lower birth weights (*p* < 0.001), shorter lengths (*p* < 0.001), smaller head circumferences (*p* < 0.001), and reduced abdominal circumferences (*p* < 0.001). These findings highlight the compromised intrauterine environment associated with preeclampsia. Neonatal health at birth was also affected, with a significantly lower mean APGAR score in the preeclampsia group (7.6 vs. 9.2, *p* < 0.001). In contrast, maternal education levels, classified using the ISCED scale, showed no significant difference between the groups (3.32 vs. 3.23, *p* = 0.450), indicating that education did not have a confounding effect when studied independently.

### 3.2. Maternal Education Levels

To better analyze and understand the effects of maternal education, we applied a stratification algorithm based on ISCED levels, as described in the study design. Additionally, these subgroups were further divided based on the preeclampsia status.

Overall, the largest proportion of patients fell into the Low Education category (ISCED 1–2), accounting for 43.5% of the population, followed by Medium Education (ISCED 3–4) at 29.4%, and High Education (ISCED 5–6) at 27.2%. This distribution indicates a higher prevalence of individuals with lower educational levels in the study population, representative of the regional geosocial environment. This trend was partially maintained when separately studying the preeclampsia and control groups, with visible discrimination, especially in the Low Education subgroup.

**Figure 1:**
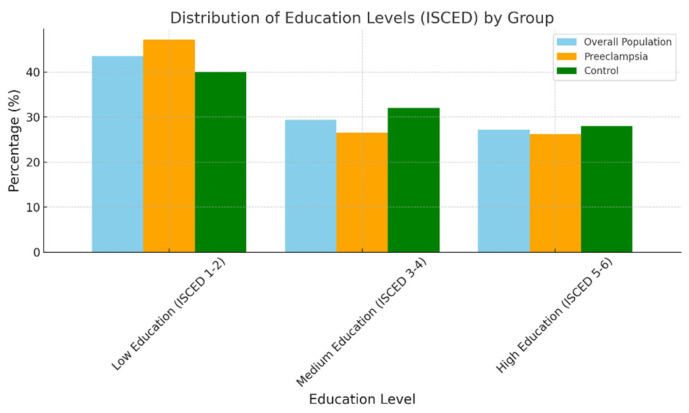
Histogram of descriptive statistics of population distribution by ISCED subgroup.

The histogram illustrates the distribution of education levels (categorized using the ISCED system) among the three groups: the overall population, individuals with preeclampsia, and the control group (Figure 1).

In the preeclampsia group, there was an increase in the share of individuals with Low Education (47.2% of the group), compared to 26.5% in the Medium Education category and 26.2% in the High Education category. This suggests that patients with a lower educational level may be overrepresented in the preeclampsia group, possibly due to factors such as healthcare accessibility or socioeconomic challenges. In contrast, the control group shows a more balanced distribution of education levels, with Low Education at 40.0%, Medium Education at 32.0%, and High Education at 28.0%. This indicates a more even spread of education levels within the control group, reflecting the expected distribution in pregnancies and being in trend with local statistics.

### 3.3. Neonatal Outcomes

Neonatal outcomes were assessed in the overall population and within the preeclamptic and control subgroups, focusing on birth weight, gestational age, and APGAR scores.

#### 3.3.1. Fetal Birth Weight

The analysis of birth weight in relation to maternal education reveals notable trends across all groups. In the overall population, fetuses from mothers with low education had a mean birth weight of 2990 g (SD = 667), which was lower than that of mothers with medium (3028 g/SD = 738) and high education (2963 g/SD = 738). These trends were accentuated in the preeclampsia group. Newborns of mothers in the low-education category had a mean birth weight of 2831.5 g (SD = 739.8). This finding aligns with the well-established link between preeclampsia and lower birth weights, as this condition is associated with restricted fetal growth and preterm delivery. In the control group, the mean birth weight in the low-education subgroup was 3164.1 g (SD = 528.4), which is closer to the typical birth weight for full-term infants. This suggests that in the absence of pregnancy complications, maternal education has a diminished influence on birth weight (Table 2).

To determine whether the difference in birth weight across education levels was statistically significant, we conducted an ANOVA. The ANOVA *p*-value for the overall population was <0.001, indicating a significant variation in birth weight among education levels. In the preeclampsia group, *p* < 0.001 further supports the influence of education on birth weight. However, in the control group, the *p*-value of 0.943 indicates no significant difference, suggesting that maternal education has a less pronounced effect on birth weight in healthy pregnancies compared to high-risk pregnancies.

**Figure 2:**
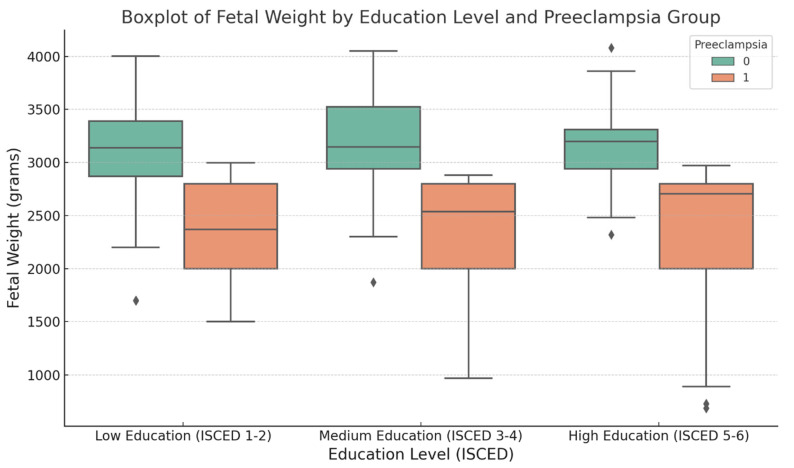
Boxplot of Fetal birth weight by preeclampsia group and education level (ISCED).

The boxplot illustrates the fetal weight distribution across the three ISCED subgroups, with data separated between the preeclampsia and control groups. The control group consistently exhibited higher fetal weights across all education levels, whereas the preeclampsia group showed a wider range of fetal weights, with a lower median than that of the control group. Additionally, the interquartile range (IQR) was broader in the preeclampsia group, particularly within the Low Education subgroup, highlighting greater variability in fetal weights within this category (Figure 2).

#### 3.3.2. Gestational Age

The analysis of gestational age in relation to maternal education points to trends across the overall population, as well as within the preeclampsia and control groups. In the overall population, newborns from mothers with low education had the lowest mean gestational age of 35.6 weeks (SD = 4.2), suggesting that lower education levels may be linked to earlier deliveries, possibly due to factors such as limited prenatal care or pregnancy complications. In contrast, mothers with medium and high education levels had babies with mean birth gestational ages of 36.7 weeks (SD = 3.4) and 36.97 weeks (SD = 3.3), respectively. This suggests that higher education may be associated with longer gestational periods, likely due to better access to healthcare and healthier lifestyles. Within the preeclampsia group, these downward trends were exacerbated, with mothers in the low-education category delivering at a mean of 33.4 weeks (SD = 4.4), reinforcing the well-established link between preeclampsia and preterm birth. For the control group, low education level was associated with a mean gestational age of 38.04 weeks (SD = 2.09) (Table 3).

We conducted an ANOVA to determine whether the difference in gestational age across education levels was statistically significant. The ANOVA *p*-value for the overall population was 0.0003, indicating a significant difference between the groups. In the preeclampsia group, the *p*-value of 0.0067 further supports the impact of education on the timing of delivery. However, in the control group, the ANOVA *p*-value of 0.107 suggests no significant difference, implying that in healthy pregnancies, education may not play a major role in determining the timing of the delivery.

**Figure 3:**
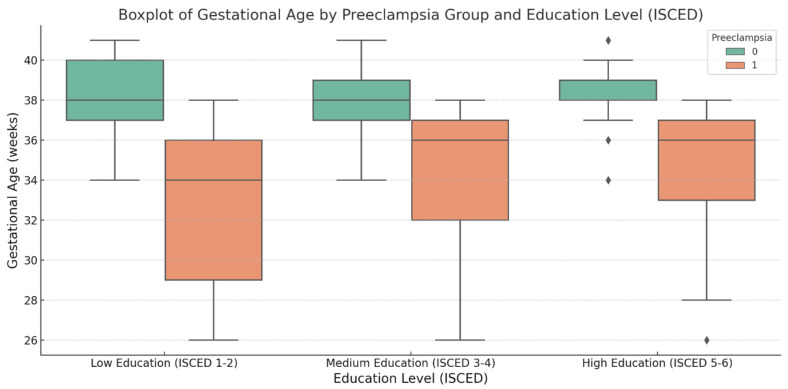
Boxplot of gestational age at birth by preeclampsia group and education Level (ISCED).

The boxplot for gestational age illustrates how delivery timing varies across the preeclampsia and control groups within the three ISCED subgroups. The control group consistently shows higher median gestational ages across all education levels. In contrast, the preeclampsia group experienced earlier deliveries, with the median gestational age in the low-education subgroup significantly lower than that in the control group, where there was greater variability in gestational age. Overall, the boxplot highlights that preeclampsia is strongly associated with earlier deliveries, especially among mothers with lower levels of education (Figure 3).

#### 3.3.3. APGAR Scores

Furthermore, the analysis of APGAR scores in relation to maternal education underlines the same trend of differences across the overall population, preeclampsia, and control groups. In the overall population, mothers with low education had a mean APGAR score of 8.3 (SD = 2.0), which was lower than those with medium and high education, whose mean scores were 8.8 (SD = 1.1) and 9.1 (SD = 1.2), respectively. These results suggest that higher education is associated with better neonatal health, as reflected in higher APGAR scores. In the preeclampsia group, the mean APGAR score was lower, with mothers with low education yielding a fetal mean of 7.8 (SD = 2.0). This is consistent with the increased risk of poor neonatal outcomes in preeclamptic pregnancies. In contrast, the control group shows higher mean APGAR scores, with low education corresponding to a mean of 8.9 (SD = 0.7), indicating that healthy pregnancies are less affected by maternal education (Table 4).

We utilized ANOVE to determine whether the differences across all three subgroups were statistically significant regarding the ISCED. The ANOVA *p*-value for the overall population was <0.001, indicating a significant difference in APGAR scores between the education levels. For the preeclampsia group, the *p*-value of < 0.001 also showed a significant effect of education on APGAR scores. However, in the control group, the *p*-value of 0.0018 suggests that education still plays a role in improving neonatal health, but the effect is less pronounced than that in the diseased group.

**Figure 4:**
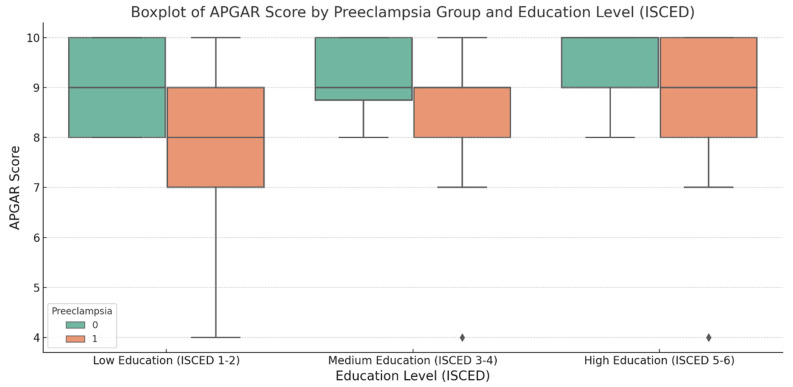
Boxplot of APGAR score by preeclampsia group and education level (ISCED).

The boxplot for APGAR scores illustrates their distribution across the preeclampsia and control groups within the three ISCED education subgroups. The control group consistently had higher median APGAR scores, indicating better neonatal health in uncomplicated pregnancies. In contrast, the preeclampsia group showed lower median APGAR scores, especially in the low-education subgroup. In addition, the plot points out that preeclampsia is associated with lower APGAR scores, particularly in those with lower education levels, which is a trend that has already been observed (Figure 4).

In summary, global ANOVA analysis revealed significant differences in birth weight, gestational age, and APGAR scores across education levels, primarily in the overall population and the preeclampsia group. For birth weight, the *p*-values were <0.001 in both the overall population and preeclampsia group, while the control group showed no significant difference (*p* = 0.943). Similarly, gestational age was significantly influenced by education in the overall population (*p* = 0.0003) and the preeclampsia group (*p* = 0.0067), whereas the control group showed no significant effect (*p* = 0.107). APGAR scores also varied significantly with education in the overall population (*p* < 0.001) and preeclampsia group (*p* < 0.001), with the control group showing a weaker but still significant effect (*p* = 0.0018). These findings show that maternal education has an impact on birth outcomes in preeclamptic pregnancies compared to uncomplicated pregnancies (Table 5).

#### 3.3.4. Low Birth Weight and Fetal Growth Restriction

A total of 25.6% of the neonates in the study had low birth weight (<2500 g). The rate of low birth weight was significantly higher in the preeclamptic group (35%) than in the control group (15%), yielding a *p*-value < 0.001 when evaluated using the chi-square test. When accounting for fetal growth restriction under the 3rd percentile, we found that 22% of neonates in the preeclamptic group were classified as such, compared to 10% in the control group (*p* < 0.001).

#### 3.3.5. Statistical Modelling

Logistic regression was performed to assess the effect of maternal education on neonatal outcomes and determine a statistical cut-off point for the ability of ISCED prediction, all adjusted for potential confounders. The results showed that maternal education was a significant predictor of birth weight in the preeclamptic group, with higher education levels associated with a lower likelihood of low birth weight (<2500 g) (OR = 0.68, 95% CI 0.47–0.97 for high vs. low education; *p* = 0.03).

**Figure 5:**
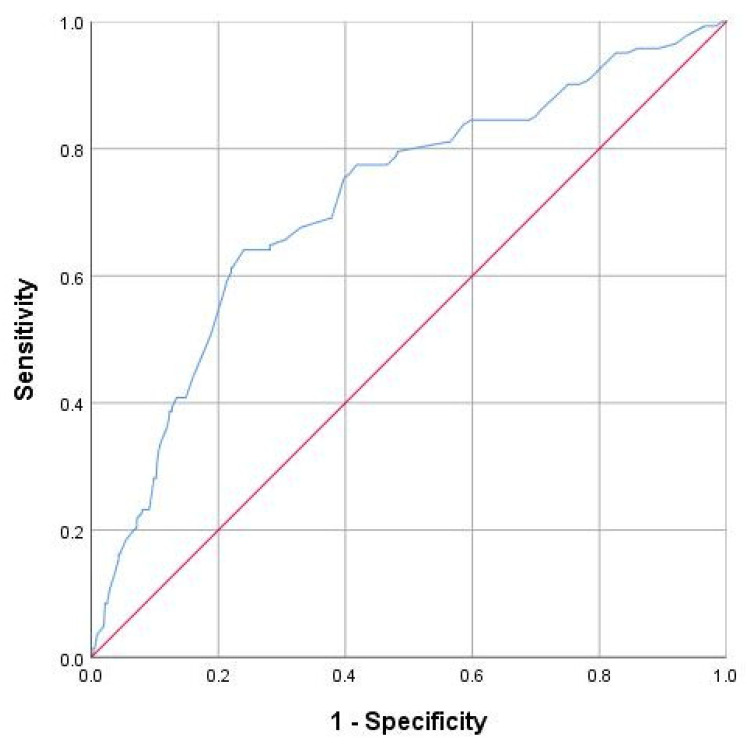
ROC of the predictive value of maternal education on fetal birth weight.

Receiver operating characteristic (ROC) analysis, adapted for gestational age, revealed that maternal education level had a moderate ability to predict low birth weight, with an area under the curve (AUC) of 0.72 (95% CI: 0.68–0.77). Considering all ISCED levels, not only our stratified subgroups, the optimal cut-off for predicting low birth weight was an ISCED level of 4 (medium education), which had a sensitivity of 0.75 and a specificity of 0.65 (Figure 5).

## 4. Discussion

### 4.1. Comparison with Existing Literature

Our findings are in concordance with those of previous studies, indicating that preeclampsia increases the risk of preterm birth, low birth weight, and altered APGAR scores. In addition, the literature also points to adverse maternal outcomes, raising both mortality and morbidity in affected individuals [15]. Specifically reflecting on maternal education, research has shown that lower education is associated with an increased risk of adverse pregnancy outcomes, which is highly impacted by healthcare access and compliance to indications and follow-up—or lack thereof [16,17]. Studies have also highlighted potential biases in APGAR scoring, suggesting that various factors can influence the score, including maternal sedation or anesthesia, congenital malformations, gestational age, and trauma. Further studies might also indicate that a lower socioeconomic status may predispose patients to a lower APGAR score based on association bias. These factors contribute to variations in APGAR scores among different populations [18,19].

Our study population revealed several important sociodemographic differences between the groups. Although maternal age and gravidity were similar between the groups, the preeclampsia group had significantly fewer viable pregnancies and lower educational attainment. This may reflect underlying disparities in health access and prenatal care engagement. These characteristics align with previous findings in similar low- and middle-income settings [15,17].

### 4.2. Potential Mechanisms and Explanations

One possible explanation for the observed influence of maternal education on neonatal outcomes is that higher education is associated with improved prenatal care adherence, better nutritional choices, and health literacy, as discussed previously [7]. Educated mothers are more likely to engage with healthcare providers, attend regular prenatal visits, and seek timely medical interventions whenever a symptom might be present. When comparing patients with a lower socioeconomic status, those collectively contribute to better neonatal outcomes, thus showing a mitigating effect of one’s education on the disease [20,21].

In well-educated patients, a tendency to receive higher APGAR scores may be due to both improved prenatal care and the perception that these patients are more compliant and better managed [19,22]. As reflected in the analysis, individuals with higher education (ISCED 5–6) are more likely to engage in regular prenatal visits, follow medical advice, and access timely interventions, all of which contribute to healthier neonatal outcomes. Thus, the mother-baby social association bias in APGAR scoring must be acknowledged. Research suggests that healthcare providers may unconsciously assign higher scores to infants born to mothers with higher education levels due to perceived better maternal engagement and health awareness [22,23]. This could lead to slight but systematic differences in neonatal assessments, potentially inflating APGAR scores when extrapolated globally. Another significant concern is the potential for racial bias, particularly in terms of skin color. Studies have indicated that lower APGAR scores are given to Black neonates than to non-Black neonates, even when controlling for other factors [24]. Future research should investigate this phenomenon further to ensure that neonatal evaluations remain both objective and equitable.

On the other hand, lower-educated patients (ISCED 1–2) may not receive consistent medical supervision throughout their pregnancies, either by choice or due to limited access to healthcare providers. As seen in the boxplot, low-educated individuals from the control group often have longer gestations, with many delivering beyond 40 weeks, possibly due to a lack of adequate prenatal care and delayed medical intervention. In contrast, higher-educated patients are less likely to exceed 39 weeks, as they are more frequently monitored, and the pregnancies are more often terminated by cesarean section [25,26]. This may also be due to local regulations, habits, and preferences. Thus, we suggest an explanation for the higher gestational age in lower-educated patients presented in our study.

In the diseased group, cesarean section is a common practice worldwide for managing preeclampsia and other related complications [27]. Early interventions observed in these patients can lead to improved outcomes, prevent risks associated with unnecessarily prolonged toxic pregnancy, and ensure safer deliveries [28].

Furthermore, the analysis highlights that lower-educated patients are also more prone to severe preterm births, especially when suffering from preeclampsia, as highlighted by the lower lows in the boxplot. This may be due to poor prenatal follow-up, which leads to late recognition of complications. These patients more often present to the hospital when the complications of either early-or late-onset preeclampsia have already become severe and are beyond a reparable state. In such cases, emergency delivery becomes necessary, leading to premature birth.

Our findings reinforce the importance of maternal education, not only as a background factor but also as a modifiable determinant of neonatal risk in high-risk pregnancies. Interventions such as educational counseling, antenatal classes, and personalized follow-up protocols may be particularly effective in improving outcomes for women with lower educational attainment.

### 4.3. Health Literacy, Access to Care, and Health Behaviors

Health literacy and access to prenatal care play pivotal roles in shaping pregnancy outcomes, particularly in high-risk pregnancies, such as those complicated by preeclampsia. Numerous studies have demonstrated that women with lower educational attainment are less likely to recognize the early symptoms of hypertensive disorders, less likely to attend scheduled prenatal visits, and less likely to comply with medical recommendations during pregnancy [8]. In contrast, women with higher levels of formal education typically exhibit better engagement with healthcare systems, more frequent antenatal check-ups, and improved adherence to preventive behaviors, such as proper nutrition, weight management, and timely reporting of alarming symptoms [7,13].

In Romania, rural and underserved populations continue to face substantial barriers to prenatal care, including geographic inaccessibility, physician shortages, and variable health insurance coverage [6]. In this context, formal education is a powerful proxy for healthcare navigation skills. Women with lower ISCED levels may not only lack awareness of the dangers of preeclampsia but may also delay care-seeking due to financial or cultural constraints. This delay can lead to the development of severe maternal complications before hospital admission, requiring urgent intervention that often compromises neonatal well-being [5].

A prospective study in Ethiopia revealed that adherence to antenatal care guidelines was strongly correlated with reduced intrapartum and postpartum complications, even after controlling for medical comorbidities [20]. Similarly, a systematic review by Nawabi et al. emphasized that low maternal health literacy is directly linked to adverse perinatal outcomes due to gaps in understanding antenatal screening, fetal movement monitoring, and medication adherence [7].

Health behaviors, such as smoking cessation, diet quality, and physical activity during pregnancy, are also strongly influenced by health education and literacy. According to Simoncic et al., low-SES women often face a complex web of behavioral and environmental stressors that reduce their capacity to engage in protective health behaviors during pregnancy [21]. Addressing this issue requires public health systems not only to expand physical access to care but also to invest in culturally sensitive educational interventions tailored to low-literacy populations.

### 4.4. Clinical and Public Health Implications

The findings of this study highlight the need for improved or newly implemented maternal education programs as part of prenatal care initiatives, depending on the resources available. Public health strategies should focus on increasing health literacy among pregnant women, particularly those with lower educational attainment [8,19]. Additionally, there is a need to revisit the APGAR scoring criteria to minimize potential biases and ensure more objective neonatal assessments [22]. Enhanced training programs for obstetric and neonatal care professionals may help reduce subjectivity in newborn assessments and promote standardized neonatal evaluation protocols, first center-wide and then beyond [28]. For example, a 2023 study investigated the effects of an immersive virtual reality (VR) training program on APGAR scoring. The findings revealed that participants in the VR group exhibited significant improvements in knowledge, learning satisfaction, self-confidence, and motivation compared to those in the physical model simulation and control groups. This suggests that immersive VR training can effectively enhance the proficiency of healthcare providers in accurately assessing Apgar scores [29].

### 4.5. Limitations and Future Research Directions

This study had several limitations, primarily due to its retrospective design, which limited the ability to infer causality [30]. Additionally, long-term neonatal outcomes were not assessed, and further research is needed to determine whether the observed effects persist beyond the immediate postnatal period [31]. Future studies should adopt a prospective design to examine the long-term impact of maternal education on child development and health outcomes. Another important area for future research is the potential bias in APGAR scoring. Studies utilizing blinded assessments of neonatal status could help determine whether our stipulated socioeconomic biases truly influence scoring decisions and whether alternative evaluation criteria could improve the objective assessment of neonates. This study confirms that preeclampsia is associated with significant neonatal risks, including lower gestational age, birth weight, and APGAR scores. Furthermore, the data suggest that maternal education may have a protective effect in preeclamptic pregnancies, positively influencing APGAR scores, birth weight, and gestational age in affected individuals. However, in healthy pregnancies (control group), its effect on birth weight and gestational age was weaker or absent, although it still played a role in neonatal well-being (APGAR score). Furthermore, it highlights the devastating effects of the disease in a socioeconomic context deprived of medical education.

## 5. Conclusions

This study provides key insights into the impact of maternal education on neonatal outcomes in pregnancies complicated by preeclampsia. Our findings reaffirm the significant adverse effects of preeclampsia on fetal development, suggesting that maternal education plays a partial statistical protective role in birth weight, gestational age, and APGAR scores. One important consideration is the potential influence of maternal socioeconomic status on neonatal assessment scores, such as APGAR, which warrants further investigation. From a clinical standpoint, these findings support the inclusion of structured maternal education interventions in prenatal care, particularly for women at an increased risk of preeclampsia. Health professionals should prioritize communication and counseling regarding early warning signs, lifestyle choices, and the importance of prenatal follow-up. In resource-constrained environments, maternal education may be a cost-effective proxy for risk stratification, helping to identify vulnerable patients who would benefit most from enhanced monitoring and support. While education cannot eliminate the biological risks of preeclampsia, it can substantially influence how patients navigate their care, ultimately improving neonatal outcomes. Future research should include prospective multicenter cohorts to investigate whether health literacy, prenatal care compliance, and socioeconomic support mediate the relationship between maternal education and neonatal outcomes in women with preeclamptic pregnancies.

## Figures and Tables

**Table 1:** Descriptive statistics of the studied variables by preeclampsia group.

Variable	Preeclampsia		Control		*p*-Value
	Mean	Range	Mean	Range	
Gestational age (weeks)	34	26–40	38	34–41	<0.001
Age of mother (years)	29.26	18–45	29.05	18–45	0.640
Number of conceptions	2.45	1–5	2.61	1–6	0.170
Number of viable fetuses carried	1.72	1–4	2.04	1–6	0.002
Fetus Weight (grams)	2806	690–4960	3175	1700–4890	<0.001
Fetus length (cm)	45.5	36.0–52.5	50.0	45.0–53.5	<0.001
Fetal head circumference	31.5	25.5–35.0	35.5	32.5–36.5	<0.001
Fetal abdominal circumference	30.5	21.5–37.5	35.5	32.5–39.5	<0.001
APGAR index	7.6	4–10	9.2	7–10	<0.001
Education Category (ISCED 1–6)	3.32	1–6	3.23	1–6	0.450

The dataset presents a statistical comparison between pregnancies complicated by preeclampsia and those in the control group, revealing significant differences in the key maternal and fetal parameters.

**Table 2:** Fetal weight stratification by ISCED and preeclampsia group.

Education Level (ISCED)	Group	N	Mean (Grams)	SD (Grams)	Min (Grams)	Max (Grams)
ISCED 1–2 (Low)	Overall	293	2990	667	1124	4896
	Preeclampsia	153	2831	739	1124	4766
	Control	140	3164	528	1700	4896
ISCED 3–4 (Medium)	Overall	198	3028	738	970	4967
	Preeclampsia	86	2828	941	970	4967
	Control	112	3181	482	1871	4050
ISCED 5–6 (High)	Overall	183	2963	738	690	4394
	Preeclampsia	85	2710	954	690	4394
	Control	98	3182	357	2318	4081

**Table 3:** Gestational age at birth stratification by ISCED and preeclampsia group.

Education Level (ISCED)	Group	N	Mean (Weeks)	SD	Min	Max
ISCED 1–2 (Low)	Overall	293	35.67	4.20	26	41
	Preeclampsia	153	33.49	4.46	26	40
	Control	140	38.04	2.09	34	41
ISCED 3–4 (Medium)	Overall	198	36.70	3.44	26	41
	Preeclampsia	86	34.78	4.06	26	40
	Control	112	38.18	1.82	34	41
ISCED 5–6 (High)	Overall	183	36.97	3.30	26	41
	Preeclampsia	85	35.14	3.85	26	40
	Control	98	38.55	1.45	34	41

**Table 4:** APGAR score stratification by ISCED and preeclampsia group.

Education Level (ISCED)	Group	N	Mean	SD	Min	Max
ISCED 1–2 (Low)	Overall	293	8.35	2.04	4	10
	Preeclampsia	153	7.82	2.04	4	10
	Control	140	8.94	0.78	8	10
ISCED 3–4 (Medium)	Overall	198	8.85	1.18	4	10
	Preeclampsia	86	8.38	1.39	4	10
	Control	112	9.21	0.82	8	10
ISCED 5–6 (High)	Overall	183	9.11	1.21	4	10
	Preeclampsia	85	8.61	1.49	4	10
	Control	98	9.54	0.66	8	10

**Table 5:** Effect of maternal education on birth outcomes across study groups (ANOVA *p*-values).

Variable	Overall Population (*p*-Value)	Preeclampsia Group (*p*-Value)	Control Group (*p*-Value)
Birth Weight	<0.001	<0.001	0.943
Gestational Age	0.0003	0.0067	0.107
APGAR Score	<0.001	<0.001	0.0018

## Data Availability

The raw data supporting the conclusions and results of this article will be made available upon request.

## Abbreviations

The following abbreviations are used in this manuscript:

ACOG	American College of Obstetricians and Gynecologists
AUC	Area Under the Curve
APGAR	Appearance, Pulse, Grimace, Activity, Respiration
CC BY	Creative Commons Attribution License
CI	Confidence Interval
CONSORT	Consolidated Standards of Reporting Trials
DOAJ	Directory of Open Access Journals
FGR	Fetal Growth Restriction
ISCED	International Standard Classification of Education
LD	Linear Dichroism
MDPI	Multidisciplinary Digital Publishing Institute
OR	Odds Ratio
ROC	Receiver Operating Characteristic
SD	Standard deviation
sFlt-1	Soluble Fms-like Tyrosine Kinase-1
SPSS	Statistical Package for the Social Sciences
TNF-α	Tumor Necrosis Factor Alpha
TLA	Three-Letter Acronym
WHO	World Health Organization
